# Study of the Combination of Self-Activating Photodynamic Therapy and Chemotherapy for Cancer Treatment

**DOI:** 10.3390/biom9080384

**Published:** 2019-08-20

**Authors:** Luís Pinto da Silva, Carla M. Magalhães, Ara Núñez-Montenegro, Paulo J.O. Ferreira, Diana Duarte, José E. Rodríguez-Borges, Nuno Vale, Joaquim C.G. Esteves da Silva

**Affiliations:** 1 Chemistry Research Unit (CIQUP), Faculty of Sciences of University of Porto (FCUP), Rua do Campo Alegre 687, 4169-007 Porto, Portugal; 2 LACOMEPHI, GreenUPorto, Faculty of Sciences of University of Porto (FCUP), Rua do Campo Alegre 687, 4169-007 Porto, Portugal; 3 Laboratory of Pharmacology, Department of Drug Sciences, Faculty of Pharmacy, University of Porto, Rua de Jorge Viterbo Ferreira 228, 4050-313 Porto, Portugal; 4 Institute of Molecular Pathology and Immunology of the University of Porto (IPATIMUP), Rua Júlio Amaral de Carvalho 45, 4200-135 Porto, Portugal; 5 I3S, Instituto de Investigação e Inovação em Saúde, University of Porto, Rua Alfredo Allen 208, 4200-135 Porto, Portugal; 6 LAQV/REQUIMTE, Department of Chemistry and Biochemistry, Faculty of Sciences, University of Porto, 4169-007 Porto, Portugal; 7 Department of Molecular Pathology and Immunology, Institute of Biomedical Sciences Abel Salazar (ICBAS), University of Porto, Rua de Jorge Viterbo Ferreira 228, 4050-313 Porto, Portugal

**Keywords:** multidrug combinations, photodynamic therapy, chemiluminescence, anticancer drug, photosensitizer, breast cancer, prostate cancer, chemotherapy

## Abstract

Cancer is a very challenging disease to treat, both in terms of treatment efficiency and side-effects. To overcome these problems, there have been extensive studies regarding the possibility of improving treatment by employing combination therapy, and by exploring therapeutic modalities with reduced side-effects (such as photodynamic therapy (PDT)). Herein, this work has two aims: (i) to develop self-activating photosensitizers for use in light-free photodynamic therapy, which would eliminate light-related restrictions that this therapy currently possesses; (ii) to assess their co-treatment potential when combined with reference chemotherapeutic agents (Tamoxifen and Metformin). We synthesized three new photosensitizers capable of self-activation and singlet oxygen production via a chemiluminescent reaction involving only a cancer marker and without requiring a light source. Cytotoxicity assays demonstrated the cytotoxic activity of all photosensitizers for prostate and breast tumor cell lines. Analysis of co-treatment effects revealed significant improvements for breast cancer, producing better results for all combinations than just for the individual photosensitizers and even Tamoxifen. By its turn, co-treatment for prostate cancer only presented better results for one combination than for just the isolated photosensitizers and Metformin. Nevertheless, it should be noted that the cytotoxicity of the isolated photosensitizers in prostate tumor cells was already very appreciable.

## 1. Introduction

Cancer is one of the biggest scourges of our time, having been responsible for 9.6 million estimated deaths in 2018 [1], second only to heart disease [2]. Despite this, there has been significant improvement on the survival rate of oncological patients. This has been attributed to multidisciplinary care, introduction of targeted therapies, and more efficient chemotherapeutic drugs [3]. However, such improvements still do not prevent therapy failure for many patients, which can be attributed to intratumoral heterogeneity or different drug resistance mechanisms [4]. Therefore, there is an increasing demand for more efficient anticancer drugs.

One of the most promising strategies for increasing the efficiency of anticancer drugs is combination therapy, in which more than one drug or modality is used to treat a single disease [5,6,7,8,9]. The combination of drugs has presented several advantages, such as better efficacy, lower toxicity, lower dosage required for achieving an equal or higher level of efficiency, and the ability to counter drug resistance [10]. Given this, it is not surprising that the number of cancer-related clinical trials focusing on combination therapy has been increasing significantly [11], with a focus on the combination of chemotherapeutic drugs with other therapeutic modalities [12]. Herein, the objective of this work is to study the potential of combining known chemotherapeutic agents with a relatively recent and promising anticancer alternative, photodynamic therapy (PDT).

PDT is considered a minimally invasive treatment and is already in clinical use for treating some types of cancer [13,14]. PDT shows also significant advantages over more conventional approaches, such as fewer side effects, high spatiotemporal precision, fast healing of healthy tissue, and a minimally invasive nature [15,16]. The therapeutic effect of PDT results from the production of reactive oxygen species (mainly singlet oxygen) upon photo-activation of a photosensitizer via irradiation of the target tissue with light of a specific wavelength [17]. However, the low penetration of light (especially UV and visible light) into biologic tissue limits this therapy to treating tumors on or just under the skin, or on the outer lining of internal organs and cavities [18,19]. Moreover, given its localized nature, PDT is also ineffective against metastatic tumors [13].

Thus, designing photosensitizers that can be activated intracellularly and selectively without an external light source is a challenging and relevant research topic. Herein, our secondary goal is the development of single-molecule photosensitizers that can be used in PDT in which activation is not dependent on photo-excitation. This aim is feasible by employing chemiluminescent substrates as photosensitizers, as chemiluminescence consists of the conversion of thermal energy into excitation energy due to a chemical reaction [20,21,22,23,24].

In fact, chemiluminescent reactions have already been tested, both in vivo and in vitro, as excitation sources for PDT systems via energy transfer from the chemiluminescent emitter to the photosensitizer [25,26,27,28,29]. For example, Mao et al. co-encapsulated bis[2,4,5-trichloro-6-(pentyloxycarbonyl)phenyl] oxalate (CPPO) and a specially designed photosensitizer TBD into pluronic F-127 and soybean oil [25]. The production of singlet oxygen was triggered by the chemiluminescent reaction of tumor H_2_O_2_ with CPPO, which photo-excited the photosensitizer TBD by chemiluminescent resonance energy transfer (CRET). Yu et al. developed a system with a similar rationale, in which tumor H_2_O_2_ initiates the chemiluminescent reaction of CPPO, which in turn also activates the photosensitizer chlorin e6 via CRET [26]. The photodynamic system developed by Yuan et al. is also based on tumor H_2_O_2_ but employs luminol instead of CPPO as the chemiluminescent system [27]. Namely, both luminol, horseradish peroxidase (HRP), and a photosensitizer, cationic oligo (*p*-phenylene vinylene) (OPV), are added to tumor cells. Luminol is then oxidized by tumor H_2_O_2_ in a reaction catalyzed by HRP, being then able to photoactivate OPV via CRET [27]. Zhang et al. also used the luminol–HRP system as a self-activating excitation source, but they included in their system semiconducting polymer dots as intermediate CRET acceptors [29]. More specifically, the chemiluminophore of the luminol reaction transfers its energy to the polymer dots, which are the ones to photoactivate the photosensitizer by fluorescence resonance energy transfer (FRET) [29]. Finally, Hsu et al. created a complex system based on *Renilla* bioluminescence [28]. More specifically, they designed fluorescent quantum dots and immobilized at their surface the *Renilla* luciferase enzyme. This luciferase catalyzes the chemiluminescent reaction of coelenterazine. The authors also synthesized micelles loaded with the photosensitizer Foscan [28]. Tumor destruction was then achieved by co-incubation of tumor cells with the luciferase-immobilized quantum dots, coelenterazine, and the Foscan-loaded micelles, due to CRET from the coelenterazine–luciferase complex to the quantum dots, followed by FRET from the quantum dots to Foscan [28].

However, such systems do not appear to be practical for use in a clinical environment, as the chemiluminescent substrates and catalyst/co-factor must be present in the same microenvironment to generate the required light emission. However, it is not trivial to ensure that all components are delivered to the same cellular region without reacting during this process. The photosensitizer should be present in the same region as the chemiluminescent emitter, as energy transfer efficiency is inversely proportional to the donor-acceptor distance. Even if all of these conditions are met, the efficiency of the global process would be dependent on various sequential steps, which is bound to decrease the efficiency and reproducibility of PDT. Finally, the main advantage of PDT is its selectivity [13,14]. However, this advantage is lost if light excitation is replaced by a chemiluminescent system without tumor-selectivity.

To overcome these problems, we developed three single-molecule photosensitizers with potential for intracellular and tumor-selective self-excitation via a chemiluminescent process triggered by a cancer marker, and the production of singlet oxygen by being chemiexcited directly to triplet excited states without requiring activation by energy transfer. Given this, these molecules can function as both the excitation source and the photosensitizer itself, without requiring any external light source and, thereby eliminating the light-related restrictions regarding tumor size and localization that PDT currently presents [13,14,18,19].

**Figure 1:**
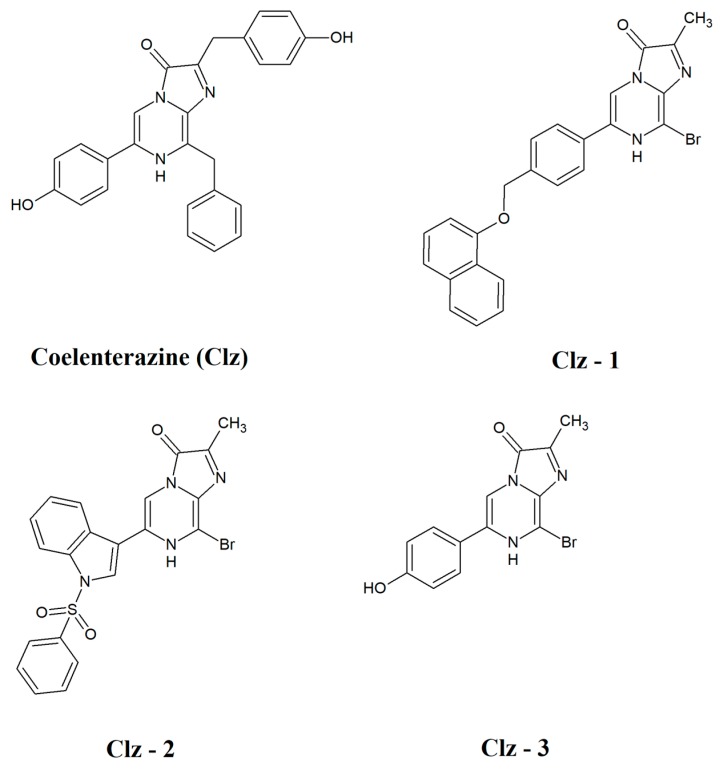
Chemical structures of natural Coelenterazine (Clz) and the three Coelenterazine derivatives here synthesized.

The new photosensitizers are based on Coelenterazine (Figure 1), a chemiluminescent imidazopyrazinone substrate found among diverse marine organisms [17,23]. More importantly, Coelenterazine is a probe for superoxide anion [30,31,32], given that the reaction with this radical leads to chemiluminescence without the need for any catalyst and/or co-factor. This process is advantageous, as tumor cells are under oxidative stress mainly due to the overexpression of superoxide anion [33,34]. Thus, the three new Coelenterazine-based photosensitizers would be just activated by a cancer marker (superoxide anion), making them intrinsically tumor-selective. Moreover, by using this type of system, only the photosensitizer is needed to be added to the patients, which eliminates the difficulty of delivering several reaction components to the tumor. It should be noted that the proposed PDT system does not consists of the simple production of reactive oxygen species, but of superoxide anion → singlet oxygen exchange. This exchange should produce oxidative damage able to bypass cell defenses, given that superoxide is a main target of the cell antioxidant machinery while singlet oxygen is not [35].

In this study, these new photosensitizers will be tested for potential anticancer applications in vitro toward breast and prostate cancer, which were responsible for about 35% of all cancer-related deaths in 2018. More specifically, we will test both their individual anticancer activity and their cytotoxic potential when combined with the reference drugs Tamoxifen—which is a common chemotherapeutic agent for breast cancer [36]—and Metformin, a chemotherapeutic drug used for treatment of prostate cancer [37].

## 2. Materials and Methods

### 2.1. Synthesis of Coelenterazine-Based Photosensitizers

The new photosensitizers were synthesized according to previous literature with some modifications [38]. The synthesis process is described in detail in the Supplementary Materials. Briefly, three different 3-bromopyrazinamine derivatives (1 equiv, previously prepared, see Supplementary Materials) were dissolved in ethanol (3 mL/mmol) and kept under argon atmosphere. To the reaction mixture were added methylglyoxal (2 equiv) and concentrated hydrochloric acid (5 equiv). The mixture was stirred for 4 h at 80 °C. After cooling to room temperature, the solvent was evaporated under reduced pressure and the residues was washed with AcOEt and ether, affording the corresponding Coelenterazine derivatives (Clz–1, Clz–2 and Clz–3) as yellow-brownish powders.

8-bromo-2-methyl-6-(4-((naphthalen-1-yloxy)methyl)phenyl)imidazo[1,2-α]pyrazin-3(7H)-one (**Clz-1**). Yield: 78%. ^1^H-RMN (MeOD, 400 MHz) δ ppm: 8.85 (s, 1H), 8.39 (d, 1H), 8.18 (s, 1H), 8.16 (d, 2H), 7.77 (s, 1H), 7.76 (d, 2H), 7.71 (s, 1H), 7.67 (d, 1H), 7.60 (t, 1H), 6.76 (d, 1H), 5.42 (s, 2H), 2.57 (s, 3H).

8-bromo-2-methyl-6-(1-(phenylsulfonyl)-1H-indol-3-yl)imidazo[1,2-α]pyrazin-3(7H)-one (**Clz-2**). Yield: 79%. ^1^H-RMN (MeOD, 400 MHz) δ ppm: 8.82 (s, 1H), 8.46 (s 1H), 8.20 (d, 1H), 8.11 (d, 1H), 8.07 (d, 2H), 7.67 (t, 1H), 7.57 (t, 2H), 7.44 (m, 2H), 2.58 (s, 3H).

8-bromo-6-(4-hydroxyphenyl)-2-methylimidazo[1,2-α]pyrazin-3(7H)-one (**Clz-3**). Yield: 67%. ^1^H-RMN (MeOD, 400 MHz) δ ppm: 8.69 (s, 1H), 7.94 (d, 2H), 6.94 (d, 2H), 2.57 (s, 3H).

### 2.2. Photophysical Properties and Detection of Singlet Oxygen

Chemiluminescent assays were performed in a homemade luminometer using a Hamamatsu HC135-01 photomultiplier tube. All reactions took place at ambient temperature at least in sextuplicate. The chemiluminescent reactions were carried out in methanol and were initiated by the injection of the Coelenterazines solutions in methanol into an assay tube containing different amounts of potassium superoxide, resulting in a final volume of 500 μL and a concentration of chemiluminescent substrate of 10 μM. The light was integrated and recorded at 0.1 intervals.

Fluorescence spectra were measured with a Horiba Jovin Fluoromax 4 spectrofluorometer, with an integration time of 0.1 s. Slit widths of 5 nm were used for both the excitation and emission monochromators. The fluorescence spectra of the three Coelenterazine-based photosensitizers were obtained in 500-μL methanol solutions. The concentrations were always of 20 μM.

Detection of singlet oxygen was made by measuring the fluorescence quenching of 50 μM 9,10-Anthracenediyl-bis(methylene)dimanolic acid (excitation at 380 nm, emission at 407 nm), or ABDA [39], in 500 μL of DMSO–phosphate buffer pH 7.4 (75 mM) with increasing concentrations of the Coelenterazines.

### 2.3. Cell Lines and Reagents

The human prostate cancer cell line, PC-3, and the breast cancer cell line, MCF-7, were purchased from ATCC (Manassas, VA, USA). Cells were cultured in Dulbecco’s Modified Eagle’s Medium (DMEM) supplemented with 10% fetal bovine serum (FBS), 1% antibiotics/antimycotic (complete medium), and incubated in a 5% CO_2_ incubator at 37 °C. Tamoxifen was purchased from Tocris (Bristol, UK). Metformin and MTT reagent were purchased from Sigma-Aldrich (St Louis, MO, USA). Fetal bovine serum, DMEM medium, calcium, magnesium-free phosphate-buffered saline (PBS), and antibiotics/antimycotics were purchased from Merck Millipore (Burlington, MA, USA).

### 2.4. Cell Treatment

Prior to each treatment, PC-3 and MCF-7 cells were seeded in 96-well plates in triplicate with a density of 5000 cells/well. To test the independent effects of the new photosensitizers, cells were left either untreated (exposed only to vehicle: MeOH at a maximum final concentration of 0.1% *v/v*), or treated with each compound at 0.1, 1, 10, 25, 50, and 75 µM, for 24 h. The dose of metformin and tamoxifen necessary for the 50% inhibition (IC_50_) of the proliferative activity of PC-3 and MCF-7 cells, respectively, was also determined: MCF-7 and PC-3 cells were treated with 0.1, 1, 10, 20, 50, 75, and 100 µM of tamoxifen and metformin, respectively, for 24 or 72 h.

To assess the effects of the co-treatment with the photosensitizers and each antineoplastic (metformin for PC-3 or tamoxifen for MCF-7), cells in each concentration of photosensitizers were treated with the determined IC50 dose of metformin (1.27 µM) or tamoxifen (2.22 µM) for 24 or 72 h.

### 2.5. Cell Viability Assay

In the preliminary experiment, cells were treated with all photosensitizers separately at the various concentrations for 24 h as above described. Also, MCF-7 was treated with tamoxifen, and PC-3 cells were treated with metformin with the determined IC50 dose. In subsequent experiments, the photosensitizers were combined with tamoxifen or metformin, for MCF-7 and PC-3 cells, respectively, as already described. After cell treatment, the MTT assay was used for the assessment of cell viability. Cells were incubated with 100 µL of 0.5 mg/mL MTT solution for 3 h, protected from light. After replacing the MTT solution with 100 µL of DMSO, the plate was shaken for 10 min to solubilize the formazan crystals, and the absorbance of each well was measured at 570 nm (Sinergy HT, Biotek Instruments Inc., Winooski, VT, USA). Fractional cell inhibition was then calculated by subtracting the viability of the treated cells from the viability of the control cells, and normalized by the viability of the control cells.

### 2.6. Statistical Analyzes

Data were analyzed using GraphPad Prism software. All data were assayed in three independent experiments and the results are presented as mean ± SEM. A Student’s *t*-test and one-way ANOVA test followed by a Dunnett’s multiple comparison were used to determine the differences between control and treatment groups. A *p*-value less than 0.05 was considered statistically significant.

## 3. Results and Discussion

### 3.1. Photophysical and Photochemical Characterization of the Photosensitizers

The light-free activation of the new photosensitizers (Figure 1) is based on the direct production of triplet states via the Coelenterazine chemiluminescent reaction. However, this system is known for the efficient production of excited singlet states [21,23,40,41,42]. Thus, changing the spin of the chemiluminescent product from singlet to triplet is required. Our previous theoretical studies revealed that despite the efficient production of excited singlet states, there is also a potential pathway for intersystem crossing during this type of chemiluminescent reaction [43,44,45]. Thus, it is expected that the production of triplet states is possible if the efficiency of intersystem crossing is increased. One of the known approaches to reach this goal is the addition of heave atoms, given that they increase the rate of intersystem crossing [46]. Therefore, the three new Coelenterazines were designed with the presence of a Br heteroatom (Figure 1).

**Figure 2:**
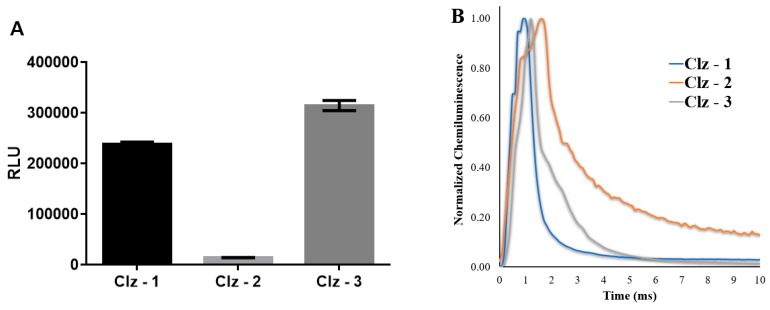

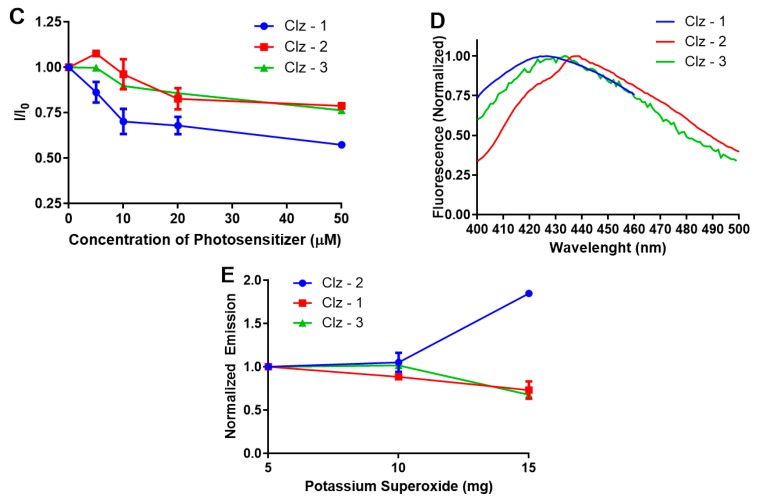
(**A**) Chemiluminescence output (in relative light units (RLU)) of Clz–1, Clz–2, and Clz–3 in methanol upon addition of 5 mg of potassium superoxide. (**B**) Normalized chemiluminescence emission, as a function of time (ms), for the three Coelenterazine derivatives in methanol upon addition of 5 mg of potassium superoxide. (**C**) Effect exerted on the fluorescence (I/I_0_) of ABDA, as a function of the concentration of the three Coelenterazine derivatives. (**D**) Fluorescence spectra of Clz–1, Clz–2, and Clz–3 in methanol. (**E**) Normalized chemiluminescence emission of Clz–1, Clz–2, and Clz–3 in methanol, as a function of the added amount of potassium superoxide.

The new photosensitizer molecules were found to fluoresce (Figure 2) with emission of blue light (upon excitation at 280 nm), with similar wavelength maxima (between 420 and 440 nm). The ability of superoxide anion to initiate the chemiluminescent reaction of the photosensitizers was demonstrated by measuring their chemiluminescent output after adding a 10-μM solution of them in methanol to 5 mg of potassium superoxide (a source for superoxide anion). In fact, all three photosensitizers were able to emit light (Figure 2), confirming their reactivity towards this reactive oxygen species. Nevertheless, Clz–3 was the molecule with the most efficient chemiluminescence, with Clz–1 also presenting appreciable chemiluminescence. By comparison, light-emission by Clz–2 was quite negligible. Furthermore, our analysis demonstrates that the chemiluminescent reaction of these photosensitizers followed a “flash” profile (Figure 2). Interestingly, the emission intensity of the photosensitizers was dependent on the amount of potassium superoxide used (Figure 2). However, only the emission of Clz–2 increased with an increase of potassium superoxide, while the emission of both Clz–1 and Clz–2 decreased. These results suggest that Clz–1 and Clz–2 might be excessively oxidized by superoxide anion above a certain threshold.

The production of singlet oxygen as the result of the chemiluminescent reaction of the three photosensitizers was confirmed by fluorescent assays using ABDA as a selective singlet oxygen sensor (Figure 2) [39]. ABDA was photobleached by singlet oxygen, which allows the monitoring of this reactive oxygen species by measuring the fluorescence quenching of ABDA. Interestingly, the photosensitizers were able to quench the fluorescence of ABDA, especially at higher concentrations. Of the three, Clz–1 presented the best results, achieving quenching values higher than 25%. Clz–2 and Clz–3 showed more moderate results.

### 3.2. In Vitro Cytotoxicity of the Photosensitizers

**Figure 3:**
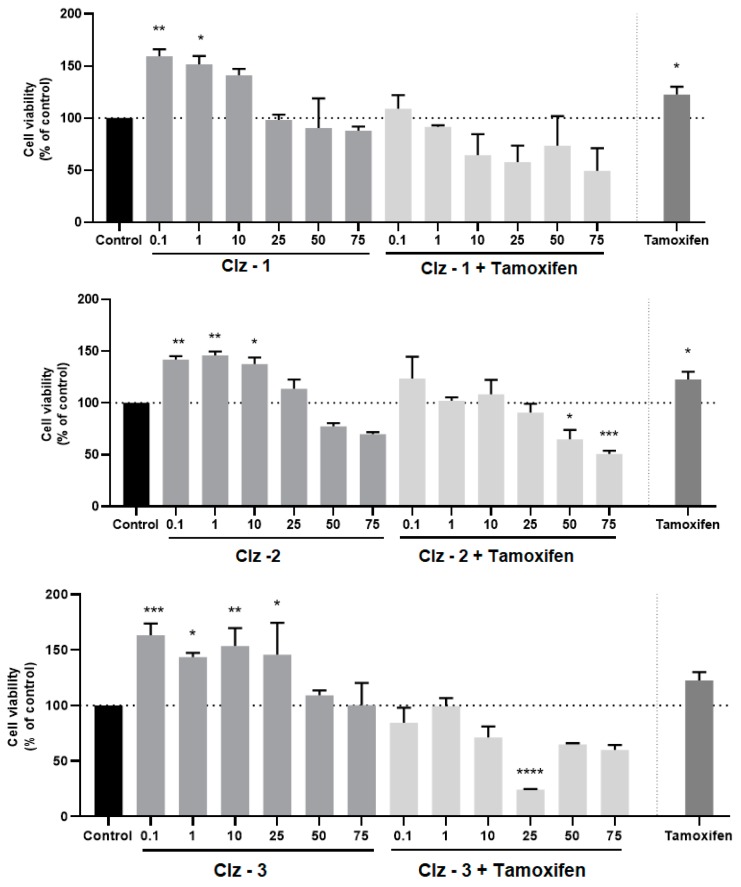
Relative viabilities of MCF-7 cells after 24-h incubations of various concentrations of either just Clz–1, Clz–2, and Clz–3, or each Coelenterazine derivative combined with Tamoxifen (2.21 μM). * Significantly different from control (p <0.05).

**Figure 4:**
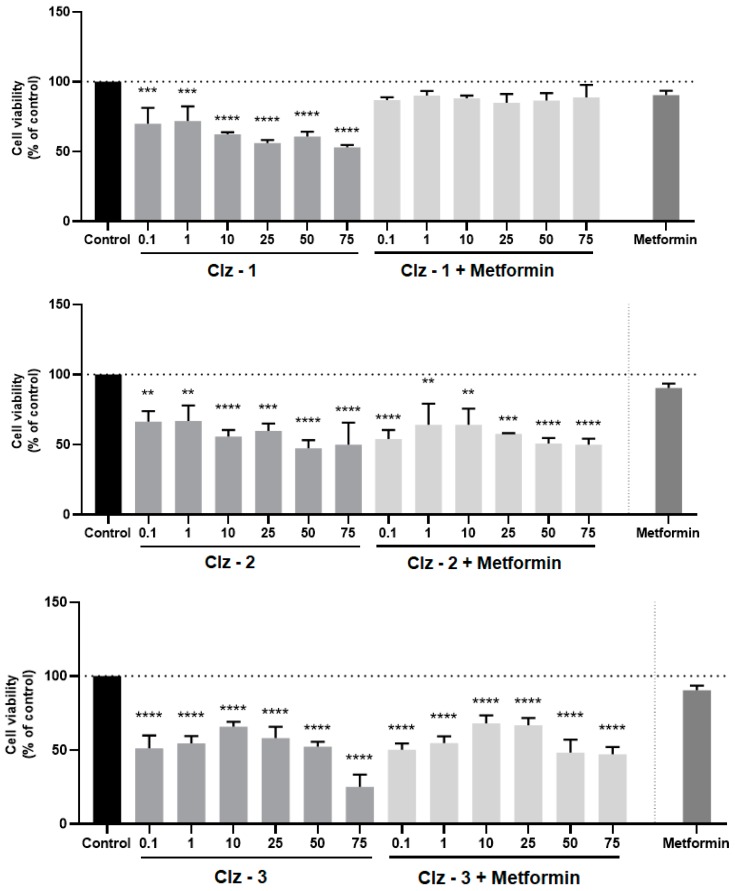
Relative viabilities of PC-3 cells after 24-h incubations of various concentrations of either just Clz–1, Clz–2, and Clz–3, or each Coelenterazine derivative combined with Metformin (1.27 μM). * Significantly different from control (p <0.05).

**Figure 5:**
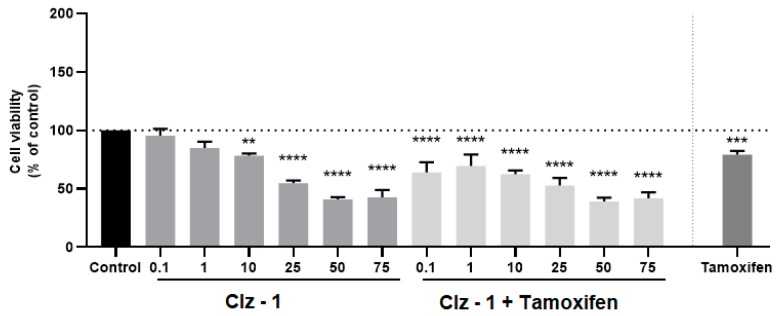

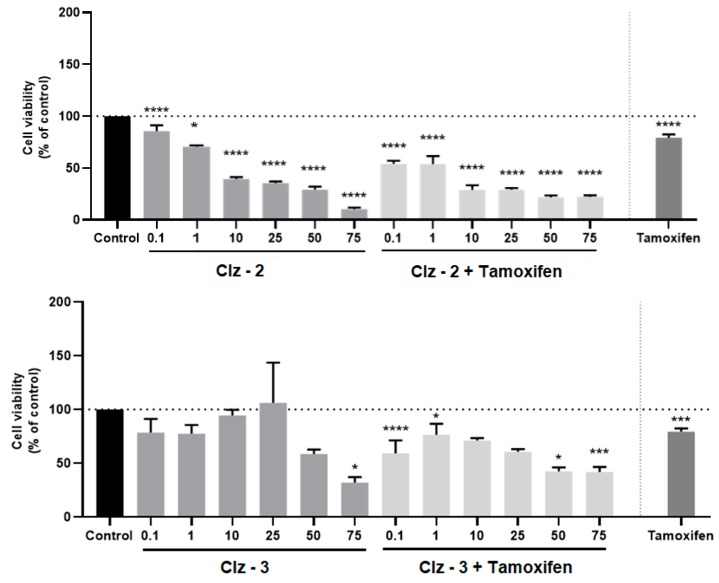
Relative viabilities of MCF-7 cells after 72-h incubations of various concentrations of either just Clz–1, Clz–2, and Clz–3, or each Coelenterazine derivative combined with Tamoxifen (2.21 μM). * Significantly different from control (p <0.05).

**Figure 6:**
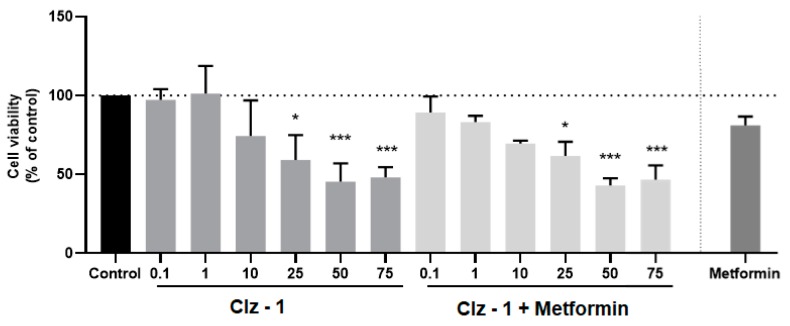

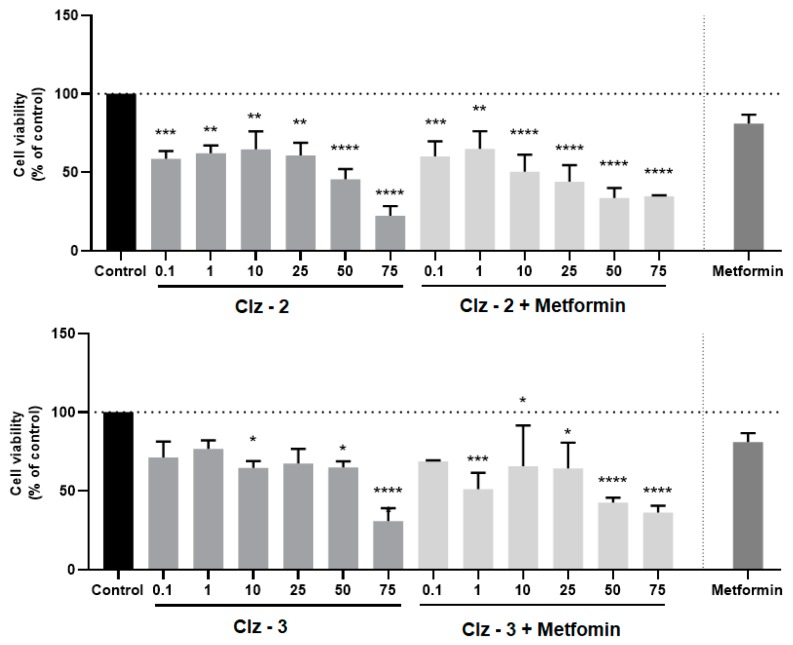
Relative viabilities of PC-3 cells after 72-h incubations of various concentrations of either just Clz–1, Clz–2, and Clz–3, or each Coelenterazine derivative combined with Metformin (1.27 μM). * Significantly different from control (p <0.05).

The cytotoxicity of the photosensitizers toward breast and prostate cancer was assessed in vitro by incubating the three Coelenterazine derivatives with MCF-7 and PC-3 tumor cell lines, respectively (Figure 3, Figure 4, Figure 5 and Figure 6). The IC_50_ values were also calculated and can be found in Table 1.

For breast cancer, the photosensitizers required a longer incubation time, given that the results were better for 72 h of incubation. This was in line with the IC_50_ values, which decreased significantly with incubation time. Interestingly, upon 72 h, the IC_50_ values of Clz–2 were three times lower than the those presented by the reference drug (Tamoxifen). Furthermore, the IC_50_ value of Clz–1 was quite similar to that presented by Tamoxifen. As for prostate cancer, the three photosensitizers were better than the reference drug (Metformin) after 24 h of incubation. That is, these new molecules were very active for a short amount of time for prostate cancer cells.

Given this information, our results demonstrate that these new photosensitizers do possess cytotoxicity toward tumor cell lines, in some cases with better results than the reference drugs. Thus, these new Coelenterazine derivatives possess the potential for being used in light-free PDT, which could overcome the limitation that this anticancer therapy currently possesses. The toxicity of all compounds was also assessed for non-tumorigenic epithelial cell lines (MCF-10A), but no toxicity was observed (data not shown), which supports the possibility of the intrinsic tumor-selectivity of these compounds.

### 3.3. Co-Treatment Studies

Having determined that the new Coelenterazine derivatives possess cytotoxic activity toward breast and prostate tumor cell lines, we proceeded to studying the effects of the co-treatment of the photosensitizers and each antineoplastic (metformin for PC-3 or tamoxifen for MCF-7). More specifically, the cells were treated with different concentrations of each photosensitizer, along with the determined IC_50_ dose of metformin (1.27 µM) or tamoxifen (2.22 µM) for 24 h. These results can be found in Figure 3, Figure 4, Figure 5 and Figure 6.

Of all the studies, the co-treatment was more efficient for breast tumor cell line and for a 24-h incubation period (Figure 3, Figure 4, Figure 5 and Figure 6). Clearly, the higher effects were for concentrations higher than 10 μM. Quite encouraging is the fact that the co-treatment presented better results than just the individual photosensitizers, or even just Tamoxifen. Furthermore, it was also seen that increasing incubation time led to better results for the co-treatment, in line with the results for IC_50_ measurements for the individual compounds (Table 1). The result of this is that, upon 72 h of incubation, the cytotoxicity assays for all combinations were better than the isolated photosensitizers and reference drug. In fact, higher combination efficiency was seen in this incubation period and this cell line (breast cancer).

On the contrary, co-treatment was not very effective for prostate tumor cell lines (Figure 3, Figure 4, Figure 5 and Figure 6) upon 24 h of incubation, when compared with the isolated photosensitizers and Metformin. At most, the co-treatment option could be slightly better if compared with the response obtained by just Metformin. Nevertheless, it should be noted that the three photosensitizers already presented quite good individual responses. Finally, increasing the incubation time to 72 h did improve the co-treatment profiles of Clz–3 and Metformin. In fact, in the two highest concentrations of photosensitizer, the Clz–3/Metformin combination provided better results than the isolated photosensitizers or reference drug.

## 4. Conclusions

In conclusion, we synthesized three new Coelenterazine derivatives with cytotoxic activity toward breast and prostate tumor cell lines. This toxicity is attributed to the superoxide anion (overexpressed in tumor cells)-induced chemiluminescent reactions of these molecules, which leads to the formation of reactive singlet oxygen. Thus, these new molecules have the potential to be used as self-activating photosensitizers in light-free PDT, which could overcome the light-related restrictions that this anticancer therapy currently possesses.

Furthermore, the co-treatment potential of these molecules was analyzed when combined with reference chemotherapeutic agents: Tamoxifen for breast cancer, and Metformin for prostate cancer. For breast cancer, co-treatment is a good option for both 24- and 72-h incubation periods, as it presents better cytotoxicity results for all combinations than for all individual Coelenterazines and even Tamoxifen. On the contrary, co-treatment does not provide significantly better results than the ones presented by the individual Coelenterazines and the reference drug (Metformin) for prostate cancer upon 24 h of incubation. Nevertheless, increasing the incubation time resulted in the combination of one of the Coelenterazine-based molecules and Metformin presenting better results than just the isolated Coelenterazines and Metformin for prostate tumor cell line.

## 5. Patents

Patent PPP59213 (Pending): Chemiluminescent imidazopyrazinone-based photosensitizers with available singlet and triplet excited states.

## Figures and Tables

**Table 1:** Cytotoxicity of the drugs Metformin and Tamoxifen, as well as Clz–1, Clz–2, and Clz–3, in PC-3 and MCF-7 tumor cell lines (24 and 72 h treatment). IC_50_ (μM) values are given as mean of 6 experiments.

	MCF-7 IC_50_^a^		PC-3 IC_50_^a^	
	24 h	72 h	24 h	72 h
Metformin	N.D.	N.D.	1.270 ± 0.416	0.813 ± 0.261
Tamoxifen	2.219 ± 0.194	11.07 ± 0.02	N.D.	N.D.
Clz–1	>100	12.18 ± 0.06	0.048 ± 0.426	12.11 ± 0.15
Clz–2	47.31 ^b^	3.00 ± 0.08	0.388 ± 0.459	1.647 ± 0.366
Clz–3	>100	49.59 ^b^	0.530 ± 0.525	3.949 ± 0.362

^a^ IC_50_ denotes a half-maximal inhibitory concentration; ^b^ Approximate estimation due to insufficient data points; N.D.: not determined.

## Supplementary Materials

The following are available online at https://www.mdpi.com/2218-273X/9/8/384/s1, detailed synthetic methodologies.
