# Supplementary material for: Study of the Combination of Self-Activating Photodynamic Therapy and Chemotherapy for Cancer Treatment
Source: Biomolecules. 2019 Aug 20;9(8):384. doi: 10.3390/biom9080384 (PMC6722738; doi:10.3390/biom9080384)
Supplement: Supplementary file 1 [file biomolecules-09-00384-s001.pdf]

# Supplementary material

**Steps to Clz - 3**

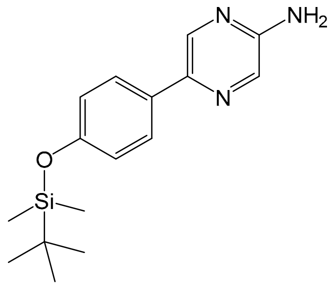


5-(4-((*tert*-butyldimethylsilyl)oxy)phenyl)pyrazin-2-amine

2-amino-5-bromopyrazine (0.13g, 0.79 mmol)) and 4-(tert-butyldimethylsilyloxy)phenylboronic acid (0.3g, 1.19 mmol) were dissolved in toluene (3mL) and stirred at room temperature. Ethanol (0.6 mL) and 1 M $Na_2CO_3$ aq. (1.2 mL) were added to the reaction mixture. After vacuum deaeration and argon gas protection, bis-(triphenylphosphine) palladium (II) chloride (5.5% of 2-amino-5-bromopyrazine) in 0.5 mL of toluene was added to the solution, and the mixture was deaerated again and stirred for 2 hours at 105 °C under argon atmosphere. The progress of the reaction was monitored by TLC. After cooling to room temperature, the solution was filtered through a Celite pad to remove the palladium catalyst. The solution was extracted with ethyl acetate, and the brown organic phase was washed with water and brine, dried over $Na_2SO_4$ and evaporated. The resulting residue was purified by silica gel column chromatography using hexane /ethyl acetate 2/1, v/v, affording 223 mg (93%) as a yellow solid. Rf: 0.20. $^1$H-RMN (CDCl3, 400 MHz) δ ppm: 8.39 (d, 1H), 8.03 (d, 1H), 7.74 (d, 2H), 6.91 (d, 2H), 4.61 (s, 2H), 1.00 (s, 9H), 0.22 (s, 6H).

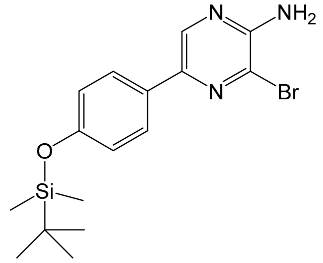


3-bromo-5-(4-((*tert*-butyldimethylsilyl)oxy)phenyl)pyrazin-2-amine

To a cooled solution of 5-(4-((tert-butyldimethylsilyl)oxy)phenyl)pyrazin-2-amine (0.1 g, 0.33 mmol) in DMF (1 mL) N-Bromosuccinimide (0.059 g, 0.33 mmol) was added in apportion at 0°C and the resulting mixture was stirred at r.t for 2 h. The reaction solution was washed with water and extracted with ethyl acetate. The organic layer was dried over

$Na_2SO_4$ anhydrous, filtered and the solvent was removed under reduced pressure. The crude product thus obtained was purified using silica gel column chromatography (hexane/ EtOAc = 5:1). Yield: 63 mg (50%). Rf: 0.46. **$^1$H-RMN** ($CDCl_3$, 400 MHz) δ ppm: 8.33 (s, 1H), 7.74 (d, 2H), 6.90 (d, 2H), 4.99 (s, 2H), 0.99 (s, 9H), 0.22 (s, 6H).

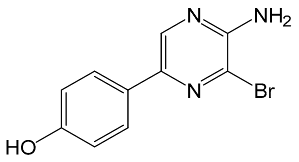


4-(5-amino-6-bromopyrazin-2-yl)phenol

3-bromo-5-(4-((tert-butyldimethylsilyl)oxy)phenyl)pyrazin-2-amine (protected compound) was dissolved in THF at 0°C and TBAF was added dropwise into the solution and the mixture was stirred for 10 minutes. Then, the reaction mixture stirred at r.t for 3 hours. After that, the solution was poured into water extracted with ethyl acetate, and dried over $Na_2SO_4$ anhydrous. The residue was purified by acid-base extraction affording 4-(5-amino-6-bromopyrazin-2-yl)phenol as the deprotected compound. Yield: 26 mg (62%). **$^1$H-RMN** ($CDCl_3$, 400 MHz) δ ppm: 8.32 (s, 1H), 7.76 (d, 2H), 6.90 (d, 2H), 5.07 (s, 2H).

**Steps to Clz - 2**

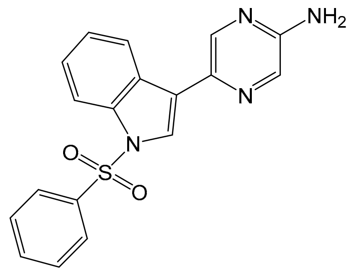


5-(1-(phenylsulfonyl)-1*H*-indol-3-yl)pyrazin-2-amine

2-amino-5-bromopyrazine (0.23g, 1.32 mmol)) and 1-(phenylsulfonyl)-3-indolylboronic acid (0.3 g, 1.19 mmol) were dissolved in toluene (7mL) and stirred at room temperature. Ethanol (2.3 mL) and 1 M $Na_2CO_3$ aq. (2 mL) were added to the reaction mixture. After vacuum deaeration and argon gas protection, bis-(triphenylphosphine) palladium (II) chloride (5.5% of 2-amino-5-bromopyrazine) in 0.5 mL of toluene was added to the solution, and the mixture was deaerated again and stirred for 2 hours at 110 °C under argon atmosphere. The progress of the reaction was monitored by TLC. After cooling to room temperature, the solution was filtered through a Celite pad to remove the palladium catalyst. The solution was extracted with ethyl acetate, and the brown organic phase was washed with water and brine, dried over $Na_2SO_4$ and evaporated. The resulting residue was purified by silica gel column chromatography using hexane /ethyl acetate 2/1, v/v, affording 330 mg (72%) as a green solid. Rf: 0.18. $^1$H-RMN ($CDCl_3$, 400 MHz) δ ppm: 8.43 (d, 1H), 8.15 (d, 1H), 8.08 (d, 1H), 8.05 (d, 1H), 7.96 (s, 1H), 7.93 (d, 2H), 7.54 (t, 1H), 7.44 (t, 2H), 7.34 (m, 2H), 4.62 (bs, 2H).

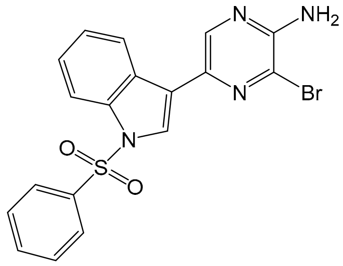


3-bromo-5-(1-(phenylsulfonyl)-1*H*-indol-3-yl)pyrazin-2-amine

To a cooled solution of 5-(1-(phenylsulfonyl)-1H-indol-3-yl)pyrazin-2-amine (0.1 g, 0.31 mmol) in DMF (1 mL) N-Bromosuccinimide (0.056 g, 0.31 mmol) was added in apportion at 0°C and the resulting mixture was stirred at r.t for 2 h. The reaction solution was washed with water and extracted with ethyl acetate. The organic layer was dried over Na2SO4 anhydrous, filtered and the solvent was removed under reduced pressure. The crude product thus obtained was purified using silica gel column chromatography (hexane/ EtOAc = 3:1) as a yellow solid (121 mg, 60 %). Rf: 0.31. $^{1}$H-RMN ($CDCl_3$, 400 MHz) δ ppm: 8.37 (s, 1H), 8.15 (d, 1H), 8.03 (d, 1H), 7.96 (s, 1H), 7.92 (d, 2H), 7.55 (t, 1H), 7.45 (t, 2H), 7.36 (m, 2H), 5.14 (bs, 2H).

**Steps to Clz - 1 = Clz - 2**

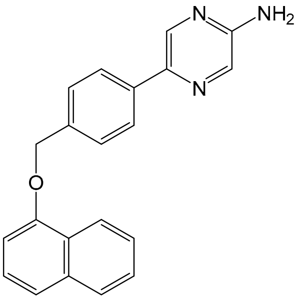


**2-amino-5-bromopyrazine (0.2 g, 1.3 mmol).**

Purified by silica gel column chromatography using hexane /ethyl acetate 1/1, v/v.

Yield: 342.1 mg (91 %). Rf: 0.36. $^{1}$H-RMN ($CDCl_3$, 400 MHz) δ ppm: 8.49 (d, 1H), 8.36 (dd, 1H), 8.06 (d, 1H), 7.93 (dd, 2H), 7.81 (dd, 1H), 7.62 (dd, 2H), 7.49 (t, 2H), 7.44 (d, 1H), 7.38 (d, 1H), 6.91 (d, 1H), 5.31 (s, 2H), 4.60 (s, 2H).

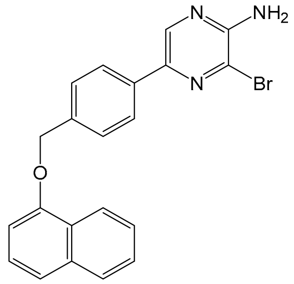


(0.2 g, 1.3 mmol). Purified by silica gel column chromatography using hexane /ethyl acetate 1/1, v/v.

Yield: 113.9 mg (46 %). Rf: 0.36. $^1$H-RMN ($CDCl_3$, 400 MHz) δ ppm: 8.43 (s, 1H), 8.37 (d, 1H), 8.18 (d, 1H), 7.90 (dd, 2H), 7.65 (dd, 2H), 7.64 (t, 1H), 7.59 (s, 1H), 7.57 (s, 1H), 7.54 (t, 1H), 6.76 (d, 1H), 5.30 (s, 2H), 5.06 (s, 2H).
